# Greenhouse gas emission associated with sugar production in southern Brazil

**DOI:** 10.1186/1750-0680-5-3

**Published:** 2010-06-17

**Authors:** Eduardo Barretto de Figueiredo, Alan Rodrigo Panosso, Rangel Romão, Newton La Scala

**Affiliations:** 1 FCAV/UNESP, Departamento de Ciências Exatas,.Via de acesso Prof. Paulo D. Castellane s/n. 14884-900, Jaboticabal, São Paulo, Brazil

## Abstract

**Background:**

Since sugarcane areas have increased rapidly in Brazil, the contribution of the sugarcane production, and, especially, of the sugarcane harvest system to the greenhouse gas emissions of the country is an issue of national concern. Here we analyze some data characterizing various activities of two sugarcane mills during the harvest period of 2006-2007 and quantify the carbon footprint of sugar production.

**Results:**

According to our calculations, 241 kg of carbon dioxide equivalent were released to the atmosphere per a ton of sugar produced (2406 kg of carbon dioxide equivalent per a hectare of the cropped area, and 26.5 kg of carbon dioxide equivalent per a ton of sugarcane processed). The major part of the total emission (44%) resulted from residues burning; about 20% resulted from the use of synthetic fertilizers, and about 18% from fossil fuel combustion.

**Conclusions:**

The results of this study suggest that the most important reduction in greenhouse gas emissions from sugarcane areas could be achieved by switching to a green harvest system, that is, to harvesting without burning.

## Background

Increasing atmospheric greenhouse gases (GHG) and its relation to human activities have pressured the productive sector to mitigate its GHG emission [1]. Developing country-specific emission factors and activity data have been a tough challenge particularly for non-Annex I countries which are recognized mostly as certain groups of developing countries that are vulnerable to the adverse impacts of climate change. Therefore the demand for assistance for non-Annex I countries to improve their inventories is likely to rise and should be effectively made [2]. Among the main practices that have caused national concern in Brazil, the harvest system is highlighted, especially in sugarcane agricultural areas, which in most regions are still based on residues burning. In contrast, the so-called green harvest, without burn, keeps large amounts of crop residues in soil surface [3].

Sugarcane residues represents 11% of the worldwide agricultural residues [4], and while sugarcane areas have increased rapidly in Brazil, few papers quantify its impact on air quality due to the land use, especially considering the burning practice [5-7] . Brazil is the biggest worldwide sugarcane grower with a 622 millions ton production (2008/2009) concentrated in 7.8 millions of hectares [8]. Those are mostly driven to ethanol (55%) and sugar (45%) derivatives, while sugarcane industrial process generate also 11.3 TWh of electric energy produced in the power plants in most of the sugarcane mills, corresponding to 3% of all electric energy consumed in the country [8]. Sugarcane is one of the world's major food-producing crops providing about 75% of the sugar for human consumption [9]. Projections indicate the biomass importance in near future that will provide up to 20% of all worldwide energy used in the end of 21 century [10]. Adding efforts to reduce emission from energy and deforestation sectors, it is also a top priority to implement innovative programs that promote mitigation in the agricultural and livestock sectors [11].

The goal of this work was to determine a scope for sugarcane mills emissions within its own boundary and quantify the GHG emissions sources related to the sugarcane production in agricultural sector in Brazil. It was applied the Intergovernmental Panel on Climate Change (IPCC) methodology [12], chapter 11, N_2_O emissions from managed soils, and CO_2 _emissions from lime and urea application, chapter 2 Generic methodologies applicable to multiple land-use categories and The First Brazilian Inventory to Mobile Combustion [13]. It was considered the total sugar production in order to determine the carbon footprint in terms of carbon dioxide equivalent (CO_2_eq) released to the atmosphere per area, ton of cultivated sugarcane and sugar produced.

## Results and Discussion

**Figure 1:**
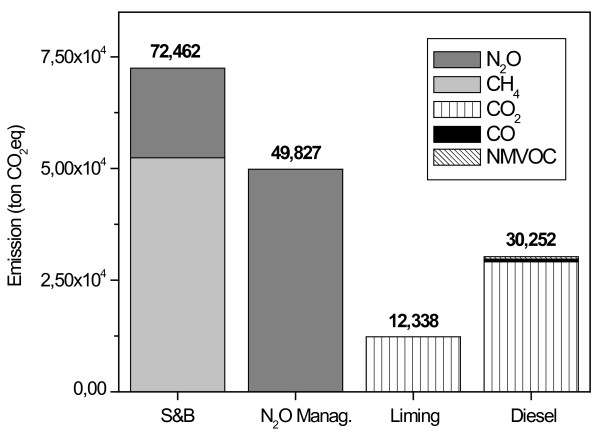
**Total GHG emissions, 2006/2007 season per source, sugarcane burning, N_2_O from managed soils, liming application and diesel use (ton CO_2_eq)**.

Figure 1 presents the partition of GHG emission for each emission source considered in this study. Based on the scenario and studied year, total company's GHG emission was 164,878 ton of CO_2_eq corresponding to 2.41 ton of CO_2_eq emitted for each cropped hectare. Some authors showed emission of 3.24 ton of CO_2_eq ha^-1 ^considering 60% of area harvested with burning practice and emission related to fertilizers, herbicides and pesticides manufacturing phase incorporated in this amount [14] while in our scope it was considered emissions related to company's boundary emissions, only. Others authors consider also emissions from the manufacture and distribution of agricultural inputs used for Brazilian sugarcane production presenting a net contribution of CO_2_from the sugarcane agro industry to the atmosphere as 3.12 ton per ha [15]. On the other hand, results have shown an average from 0.32 ton C ha^-1^yr^-1 ^accumulated in the first 20 cm depth to 1.95 ton C ha^-1^yr^-1 ^for the top 40 cm layer referring to green harvest adoption instead of burning, corresponding to as much as 7.15 ton CO_2_eq ha^-1 ^yr^-1^. This could be effectively considered a CO_2 _sequestration from atmosphere due the conversion of burned to green harvest [11], which despite the uncertain, has the potential to mitigate all GHG emission of this sector.

Residues burning accounted for 72,462 ton CO_2_eq, around 44% of total emission, equivalent to 1.21 ton of CO_2_eq for each burnt hectare, being 72% of this associated to CH_4 _emission only. In our inventory CO_2 _and CO emissions were not included as net GHG emission to atmosphere when the crop residue burning is considered. Some authors also do not compute those gases as net emission when referred to the burning practice [12,16]. CO_2 _sunk by sugarcane crops in following year compensates the amount of CO_2 _and CO (that once in atmosphere rapidly transforms in CO_2_) emitted by burning. Computing the total CO_2 _captured by photosynthesis relative to the 2006/2007 crop season with area of 68,541 ha, there is something around 5,133,212 ton of CO_2_, equivalent to 74.9 ton of CO_2 _ha^-1^sunk by sugarcane crops from atmosphere. This value is comparable to the one presented for sugarcane crops, with an amount of 107.2 ton of CO_2 _ha^-1 ^year^-1^[17].

Direct and indirect N_2_O emission due to the synthetic fertilizers use, organic composts and harvest residues caused an emission of 49,827 ton of CO_2_eq, corresponding to 30% of the total emission. Fossil fuel combustion (diesel use) and lime application contributed with 30,252 and 12,338 ton of CO_2_eq, respectively, mostly due to CO_2 _only. Substitution from diesel to biodiesel has been cited as an alternative to reduce net CO_2 _emission in this sector [17]. Also, CO_2 _emission due to diesel use could be reduced from 15 to 29% by alternative tillage systems i.e. reduced tillage, as a consequence of fuel savings [18].

**Figure 2:**
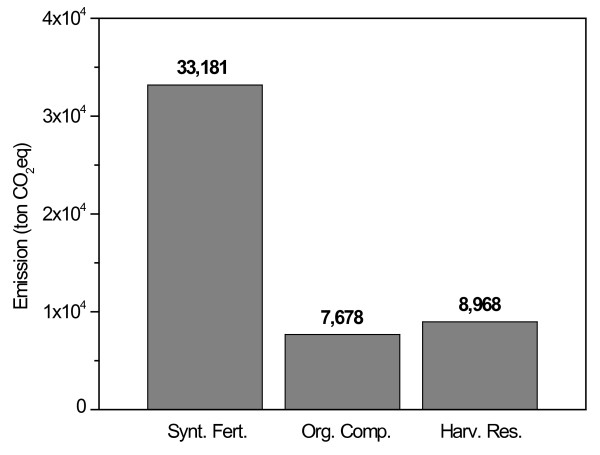
**N_2_O direct and indirect emissions from managed soils converted in CO_2_eq referring to synthetic fertilizer use, organic composts applied on soils and harvest residue**.

Figure 2 presents the partition of direct and indirect N_2_O emissions in terms of their sources. Organic fertilizers applied on soil resulted in 7,678 ton of CO_2_eq, corresponding to 15% of total N_2_O emitted in this sector. Synthetic fertilizers application resulted in 33,181 ton of CO_2_eq (67%) and it considers only the use emission, not the ones associated to the fertilizer production. The application of chemical or organic fertilizers on soil can stimulate N_2_O and NO production via nitrification (aerobic) and denitrification (anaerobic) biochemical processes [19,20]. The input of organic fertilizers to agricultural soils is considered an important source of N_2_O [21] with both chemical and organic fertilizer applications being the major sources of NH_3 _[9,22,23] . In our inventory these were some of the mainly sources of GHG emission to atmosphere, believing that such aspect is representative of sugarcane production areas.

Residues from sugarcane remained on field resulted in 8,968 ton of CO_2_eq, coming from residual N content which is converted to N_2_O through nitrification, aerobic microbial oxidation of ammonium to nitrate and denitrification process which is the anaerobic microbial reduction of nitrate to nitrogen gas (N_2_). Nitrous oxide is a gaseous intermediate in the reaction sequence of denitrification and a by-product of nitrification that is ultimately released into the atmosphere [12]. The application of nitrification inhibitors has been suggested as an option for decreasing N-fertilizer use and consequently such emission [24]. Strategies that increase N-fertilizer efficiency, reducing N_2_O emission have also been suggested by several authors [24-26] .

Table 1. presents estimations of GHG emission per kilogram of sugar produced, per hectare and per ton of sugarcane produced. According to this study each ton of sugarcane processed released 26.5 kg CO_2_eq to atmosphere, resulting 241 kg of CO_2_eq for each ton of sugar produced. Emission value for sugar beet production (Life Cycle Assessment - LCA) suggests an emission of 900 kg of CO_2_eq per ton of sugar produced [27]. LCA should be a suitable tool to assess the environmental impact associated with agricultural production [27], but this provides different methodologies to compare GHG emission in agricultural sector. In Brazil, some authors presented amounts of 222 kg CO_2_eq ton^-1 ^of sugar in the so-called organic production, without burn and without synthetic fertilizers N application [28]. That study considered emissions related to sugar transport, energy imbed in the equipments and agricultural machines and also emissions related to production of chemical supplies, resulting amounts of 34.08 kg CO_2_eq per ton of sugarcane processed, a reduction of 32% in GHG emission when compared to conventional practices that resulted in 50.44 kg CO_2_eq, considering the same scope [29].

**Table 1:** Emission ratio, kg CO_2_eq per ton of sugar, kg CO_2_eq per hectare and kg CO_2_eq per ton of sugarcane

	Emission Ratio*	
kg CO_2_eq ton^-1 ^sugar	kg CO_2_eq ha^-1^	kg CO_2_eq ton^-1 ^sugarcane

241	2,406	26.5

*kg: kilogram, CO_2_eq: Carbon dioxide equivalent, ha: hectare.

## Conclusions

Considering the studied scenario, with 87% of the total area managed with burning practice and 13% of green harvest, GHG emission ratio was 241 kg CO_2_eq ton^-1 ^of produced sugar. Each hectare of sugarcane cropped transferred to the atmosphere 2,406 kg of CO_2_eq per year. This indicate that a more sustainable agricultural production systems as conservation tillage and direct planting during the re-planting season, as well as rationalizing the N fertilizers use might be achieved to reduce GHG emissions in sugarcane areas. The total sugarcane production of 6,221,025 ton resulted in an emission ratio of 26.5 kg of CO_2_eq per ton of sugarcane processed. Considering only emissions from application and not emission from production of synthetic fertilizers N applied to soils, each kilogram used transfers to the atmosphere 6.45 kg CO_2_. Sugarcane field burning practice impacted on 1.21 ton of CO_2_eq per hectare burnt, considering only GHG net emissions. Responsible for 44% of total GHG emission, the conversion of sugarcane burning system to green harvest could reduce emissions in this sector. Considering actual production process, the company emission baseline to 2006/2007 season was 164,878 ton of CO_2_eq. The mitigation of GHG emissions from sugarcane areas could be achieved either by reducing burning and fertilization practices or using soil as a carbon sink. Applications of standardized scope, emission factors and emissions boundaries within company's activities only, show be necessary to promote comparison among companies and GHG emission reduction.

## Methods

To elaborate this work it was adopted the reference data of 2006/2007 informed by appropriated company sector, harvest period (from May 2006 to April 2007) from a sugarcane mill located in the southern Brazil, northeast region of São Paulo State, Brazil. The total sugarcane cropped area of the studied sugarcane plants in the period was 68,541 hectares (ha), resulting in a sugarcane and sugar production of 6,221,025 and 684,850 ton, respectively for both mills. In this scope we did not consider emissions related to the production of any supply (synthetic fertilizers, cement, herbicides, pesticides, steel, etc.) considering it to each company the decision to provide its own inventory.

Estimates of how much C was stored by crops in one year was calculated by considering the total sugarcane dry mass content as 53%, being 25% stalks, 12% trash, 4% green leaves and 12% roots [30]. Mill database informed an average sugarcane yield of 90.76 ton ha^-1^. To convert carbon (C) to carbon dioxide (CO_2_) it was applied the 44/12 factor (1 kilogram of carbon correspond to 3.67 kg CO_2 _captured), considering the C content in sugarcane dry matter as 42.46% [31].

The net emission was related to residues burning in the field, methane (CH_4_) and nitrous oxide (N_2_O), [12], direct and indirect N_2_O emissions from managed soils [12] and CO_2 _emissions referred to lime application. Emissions of CO_2_, carbon monoxide (CO), CH_4_, N_2_O, and NMVOC (non-methane volatile organic compounds) referred to the use of fossil fuel (total diesel consumption for all equipments and agricultural machines involved within production) were considered [13] according to Mobile Sources Brazilian National Inventory. All values were converted to CO_2 _equivalent (CO_2_eq) following the individual global warming potential for a period of 100 years for each gas, using 1 to CO_2 _[12], 3 to CO [32], 21 to CH_4_, 310 to N_2_O [12] and 3.4 to NMVOC (only to mobile combustion) [12]. Table 2. summarizes the scope considered in this work with partition in sector and emission sources.

**Table 2:** GHG emissions sources considered

Sector	Emissions Source
Agricultural	GHG* emissions due agricultural residues.
	N_2_O direct and indirect emissions from managed soils.
	CO_2 _emissions due lime application.
Mobile Combustion (Diesel vehicle)	Emissions due fossil fuel use (diesel oil).

*GHG: Greenhouse gases, N_2_O: Nitrous oxide, CO_2_: Carbon dioxide.

### Agricultural residues burning

The impact of residues burning in GHG emission took into account data from sugarcane crop varieties grown and harvested in the burnt areas only (59,820 ha). Total sugarcane yield was 5,643,786 ton in burned areas, corresponding to an average yield of 94.4 ton ha^-1^. Average values of residue per yield ratio were accounted in 19% of the varieties cropped in the burned areas indicating a residue per yield ratio of 0.205, resulting in an average of residue mass available to combustion of 19.3 ton per hectare. According to an extended review [33], the value of residues yield from different plant varieties in São Paulo state is around 19.1 ton ha^-1^. This is also similar to the amount found by other authors [34,35], of 18.2 ton of sugarcane residues per hectare. The combustion factor applied in this work was 0.80 [12].

The sugarcane residues burning result is not only CO_2 _emissions but also other GHG or precursors, including carbon monoxide (CO), methane (CH_4_), non-methane volatile organic compounds (NMVOC) and nitrogen (N_2_O, NO_x_) species [36]. Usually in the cropland and grassland areas only non-CO_2 _emissions are considered, due to the assumption that those would be counterbalanced by CO_2 _removals from the subsequent re-growth of the vegetation within one year [1]. The same applies to CO, as this is converted in CO_2 _rapidly once in atmosphere [1]. NO_x _emission was not considered as a net GHG because its global warming potential is very uncertain [1].

Different emission factors related to sugarcane residues burning have been registered in literature [37]. In this work it was used the ones suggested by IPCC, [12], Chapter 2, Generic Methodologies Applicable to Multiple Land-Use Categories (Equation 1). Those were 2.7 and 0.07 to CH_4 _and N_2_O (all values in g kg^-1 ^dry matter burnt) respectively [38].(1)

L_fire _= amount of greenhouse gas emissions from fire, tones of each GHG e.g., CH_4_, N_2_O.

A = burnt area, ha^-1^

M_B _= mass of fuel available for combustion, 19.3 ton ha^-1^.

C_f _= combustion factor, dimensionless. (default value to agricultural residues, 0.80).

G_ef _= emission factor, g kg_1 _dry matter burnt (default values 2.7 to CH_4 _and 0.07 to N_2_O)

### Direct and indirect emissions of nitrous oxide from managed soils

In this analysis, the emission sources considered were nitrogen from synthetic fertilizers and organic composts applied on soils (filter cake and vinasse), in addition to the harvest residues (Equation 2 - Direct emissions and Equation 3 and 4 - indirect emissions). In order to account for the total amount of N synthetic fertilizer applied we adopted a standard nitrogen demand from sugarcane agricultural areas in our region [39], which is around 75 kg of nitrogen (N) ha^-1 ^year^-1^. On the other hand, the amount of filter cake and vinasse applied in the production areas was informed by the company as 119,140,000 kg and 1,872,338,000 liters respectively. The N content used was 1.4 and 1.1%, for filter cake and vinasse, respectively, and those values were informed by the company, after the characterization. The N content in the filter cake was based on 25% of its dry mass, while N content of vinasse was considered as being 0.368 kg N m^-3 ^applied [40].

Equation 2 (direct emissions)(2)

Where:

N_2_O-N_*N inputs *_= annual direct N_2_O-N emissions from N inputs to managed soils, kg N_2_O-Nyr^-1^.

F_SN _= annual amount of synthetic fertilizer N applied, kg N yr^-1^.

F_ON _= Annual amounts of compost or organic N additions (filter cake and vinasse), kg N yr^-1^.

F_CR _= Annual amount of N in crop residues, kg N yr^-1^.

EF_1 _= Emission factor for N_2_O emissions from N inputs, kg N_2_O-N

(kg N input)^-1 ^= 0.01

Equation 3 (indirect emission)

N_2_O from atmospheric deposition of N volatilized from managed soils(3)

N_2_O_(ATD)_-N = annual amount of N_2_O-N produced from atmospheric deposition of N volatilized from managed soils, kg N_2_O-N yr^-1^

F_SN _= annual amount of synthetic fertilizer N applied to soils, kg N yr^-1^.

Frac _*GASF *_= fraction of synthetic fertilizer N that volatilizes as NH_3 _and NO_x_, kg N volatilized (kg of N applied)^-1^. Default value 0.10

F_ON _= annual amount of compost and other organic N additions applied to soils, kg N yr^-1 ^

Frac _*GASM *_= fraction of applied organic N fertilizer materials (F_ON_) that volatilizes as NH_3 _and NO_x_, kg N volatilized (kg of N applied or deposited)^-1^. Default value 0.20

EF_4 _= emission factor for N_2_O emissions from atmospheric deposition of N on soils and water surfaces, [kg N-N_2_O (kg NH_3_-N + NO_x_-N volatilized)^-1^. Default value 0.01

Equation 4 (indirect emission)

N_2_O from N leaching/runoff from managed soils in regions where leaching/runoff occurs(4)

N_2_O_(L)_-N = annual amount of N_2_O-N produced from leaching and runoff of N additions to managed soils in regions where leaching/runoff occurs, kg N_2_O-N yr^-1^.

F_SN _= annual amount of synthetic fertilizer N applied to soils in regions where leaching/runoff occurs, kg N yr^-1^.

F_ON _= annual amount of compost and other organic N additions applied to soils in regions where leaching/runoff occurs, kg N yr^-1^.

F_CR _= amount of N in crop residues, kg N yr^-1^.

Frac_*LEACH *_= fraction of all N added to/mineralized in managed soils in regions where leaching/runoff occurs that is lost through leaching and runoff, kg N (kg of N additions)^-1 ^default value 0.30.

EF_5 _= emission factor for N_2_O emissions from N leaching and runoff, kg N_2_O-N (kg N leached and runoff)^-1 ^Default value 0.0075.

The amount of N in harvest residue was inferred according to current methodology [12] considering sugarcane average yield for harvested without burn areas as 66.18 ton ha^-1^. As ratio residue/yield ratio is close to 0.205, 13.75 ton ha^-1 ^of above ground residues, having 1.27% of N content on it, was available for combustion [15].

Once the amount of N in each of those composts was know it is possible to infer the N_2_O emission due to the direct application of fertilizers, taking into account the emission factor given by IPCC (2006). This calculation simply converts 1% of the total N input to N_2_O emission [12].

Indirect emissions of N_2_O involves two different pathways, the first one is the volatilization of N as ammonium (NH_3_) and oxides of N (NO_x_), and the following deposit of these gases and their products NH_4_^+ ^and NO_3_^- ^in soil surface or lakes [12]. The nitrification and denitrification processes on soils transform some of these products to N_2_O returning back to atmosphere. According to the followed methodologies [12], 10% of N input of synthetic fertilizers and 20% of N input of the organic compost is volatilized and transformed into N_2_O, after nitrification and denitrification process on soils. Nevertheless, 1% of N applied on soils is transformed into N_2_O, resulting in an indirect emission effect. Leaching and runoff are also secondary pathways that could result in N_2_O emissions, in some regions. It is assumed that 30% of total N applied as synthetic and organic fertilizer and unburned residues is leached or runoff but this can also return as N_2_O by an emission factor of 0.0075, (or 0.75%)[12].

### CO_2 _emissions due lime application

The lime used during 2006/2007 season was the dolomite one CaMg(CO_3_)_2_, totalizing 25,883 ton applied in 11,423 ha (2.27 ton ha^-1^). For those it was considered an emission factor of 0.13 ton of CO_2 _per ton of dolomite lime applied [12].

### Emissions from mobile combustion

In this scope only motors powered by diesel were took into account for emission due to fossil fuel combustion (Equation 5), including company proper machinery, the transport of sugarcane stalks to the mills and all supplies within the company boundary and labor transport, totalizing 7,058,709 liters. For the third part transport (sugarcane stalks, supplies and labors) it was considered only the annual consumption of diesel (2,526,761 l) totalizing 9,585,470 liters of diesel used to calculation.

Data of diesel fleet was obtained by the company mechanization sector according to a very careful control of vehicles and its fuel consumption and traveled distance per year (kilometers year^-1^), being 25.77% of trucks and buses, 52.22% of agricultural machinery and 22.01% of cars powered by ethanol. The data from sugar transport after company's boundary were not considered. The total fleet of cars used in the production cycle is powered by the same ethanol produced by the mill, hence it's assumed that the ethanol GHG emissions (CO_2_) is reabsorbed in the next crop cycle and not accounted. The mobile sources were classified considering vehicles per category and manufacturing year, motor power and traveled distance per vehicle during the study (2006/2007 season).

Estimations of the GHG emission related to fossil fuel use in this study considered direct and indirect emissions of CO_2_, CO, CH_4_, N_2_O and NMVOC, according to the Brazilian Inventory recommendations [41]. Emission factors applied were also established (Air Control Program by Auto Motors Vehicles Pollution)/CETESB [25] in association with IBAMA (Brazilian Institute of Environment), considering type of fuel and vehicles. The methodology takes into account four steps: first it is considered data from fleet per vehicle category and second, the use of diesel, distributed by categories, and distance traveled. The next steps were to establish the emission factors, considering each vehicle, each vehicle's GHG emission per gas, and the conversion to CO_2_eq using an Excel worksheet to arrange and calculate all results and determine total emissions per vehicle and the total GHG amount. To determine the diesel emission factors it was used diesel density as 852 g liter^-1 ^and specific consume of 195 g kWh^-1^, data from Brazilian fuel. The emission factors (g liter^-1^) used in this report was established in 06 phases according to vehicles manufacturing year.(5)

E_g,t _= emission of gas *g *by fleet year/model *t*.

EF_g,t _= emission factor of gas *g*from vehicle's year *t*; (g L^-1^)

FC,t = Fuel consumption per vehicle's year *t *(liters).

## Competing interests

The authors declare that they have no competing interests.

## Authors' contributions

All authors participated in detailed discussions that led to this review paper. EBF conceived the document design and coordination, calculated the results and drafted the manuscript. ARP originally contributed to data analyses, interpretation, drafting and editing the manuscript. RR and NLSJ provided intellectual input on available data and previous analyses, and on the synthesis, presentation and interpretation needed for this review. All authors read and approved the final manuscript.

## References

[B1] Intergovernmental Panel on Climate Change - IPCC. 2007: Climate Change 2007 Solomon S, Qin D, Manning M, Chen Z, Marquis M, Averyt KB, Tignor M, Miller HL The Physical Science Basis Contribution of Working Group I to the Fourth Assessment Report of the Intergovernmental Panel on Climate Change 2007 Cambridge University Press, Cambridge, United Kingdom and New York, NY, USA 996

[B2] Umemiya C Improving GHG inventories by regional information exchange: a report from Ásia Carbon Balance and Management 2006 1 9 10.1186/1750-0680-1-9 16930465 PMC1564018

[B3] Cerri CEP Sparovek G Bernoux M Easterling WE Melillo M Cerri CC Tropical Agriculture and Global Warming: Impacvts and Mitigations Options Scientia Agricola 2007 64 n.1 83 99 10.1590/S0103-90162007000100013

[B4] IPCC Revised 1996 IPCC Guidelines for National Greenhouse Gas Inventories 1996 Bracknell

[B5] Oliveira PHF Artaxo P Pires C de Lucca S Procopio A Holben B Schafer J Cardoso LF Wofsy SC Rocha HR The effects of biomass burning aerosols and clouds on the CO_2 _flux in Amazonia Tellus 2007 59B 338 349

[B6] Cançado JED Saldiva PHN Pereira LAA Lara LBLS Artaxo P Martinelli LA Arbex MA Zanobetti A Alfesio LF Braga ALF The Impact of Sugar Cane-Burning Emissions on the Respiratory System of Children and the Elderly Environmental Health Perspectives 2006 114 5 10.1289/ehp.8485 PMC1459926 16675427

[B7] Goldemberg J Coelho ST Guardabassi P The sustainability of ethanol production from sugarcane Energy Policy 2008 36 2086 2097 10.1016/j.enpol.2008.02.028

[B8] CONAB Companhia Nacional de Abastecimento. Acompanhamento de Safra 2009 http://www.conab.gov.br/conabweb/download/safra/1cana_de_acucar.pdf Accessed in: 20 jun 2009

[B9] Food and Agriculture Organization and IFA Global estimation of gaseous emission of NH_3_, NO and N_2_O from agricultural land 2001 FAO, Rome, Italy ftp://ftp.fao.org/agl/agll/docs/globest.pdf Accessed in: 12 sep 2009

[B10] Goldemberg J Biomassa e energia Quimica Nova 2009 32 3 582 587

[B11] Cerri CC Maia SMF Galdos MV Cerri CEP Feigl BJ Bernoux Mk Brazilian greenhouse gas emissions: the importance of agriculture and livestoc Scientia Agricola 2009 66 6 831 843 10.1590/S0103-90162009000600017

[B12] Intergovernmental Panel on Climate Change - IPCC. 2006. IPCC Eggleston HS, Buendia L, Miwa K, Ngara T, Tanabe K Guidelines for National Green House Gas Inventories, Prepared by the National Greenhouse Gas Inventories Programme 2006 Japan: IGES Chapter 11, N_2_O emissions from managed soils, and CO_2 _emissions from lime and urea application. Chapter 2 Generic methodologies applicable to multiple land-use categories

[B13] Ministérioda Ciência e Tecnologia - MCT Primeiro inventário brasileiro de emissões antrópicas de gases de efeito estufa, relatórios de referência, ministério da ciência e tecnologia, emissões de gases de efeito estufa no setor energético por fontes móveis 2006 http://www.mct.gov.br/index.php/content/view/57270.html Accessed in: 10 feb.2009

[B14] Soares LH de B Alves BJR Urquiaga S Boddey MR Mitigação das emissões de Gases de Efeito Estufa pelo Uso de Etanol da Cana-de-açúcar Produzido no Brasil 2009 Seropédia: Embrapa 14 http://www.cnpab.embrapa.br/publicacoes/download/cit027.pdf (Circular Técnica, 27). Accessed in: 06 oct 2009

[B15] Oliveira de MED Vaughan BE Edward Jr Ethanol as Fuel: Energy, Carbon Dioxide Balances, and Ecological Footprint BioScience 2005 55 57

[B16] Macedo IC Seabra JEA Silva JEAR Green house gases emissions in the production and use of ethanol from sugarcane in Brazil: The 2005/2006 averages and a prediction for 2020 Biomass and Bioenergy 2008 32 582 595 10.1016/j.biombioe.2007.12.006

[B17] National Biodiesel Board http://www.biodiesel.org/Accessed in 20/09/2009. Imperial College

[B18] Koga N Tsuruta H Tsuji H Nakanoa H Fuel consumption-derived CO_2 _emissions under conventional and reduced tillage cropping systems in northern Japan Agriculture, Ecosystems and Environment 2003 99 213 219 10.1016/S0167-8809(03)00132-4

[B19] Davidson EA Microbial Production and Consumption of Greenhouse Gases: Methane, Nitrogen oxides, and Halomethanes, American Society of Microbiology Rogers JE, Whitman WB Fluxes of nitrous oxide and nitric oxide from terrestrial ecosystems 1991 Washington DC 219 235

[B20] Conrad R Murrell JC, Donovan PK Metabolism of nitric oxide in soil and soil microorganisms and regulation of flux into the atmosphere Microbiology of Atmospheric 1996 Springer-Verlag, Berlin, Heidelberg 167 203 Trace Gases, NATO ASI Series. I 39

[B21] Kroeze C Mosier A Bouwman L Closing the global N2O budget: A retrospective analysis 1500-1994 Global Biochem Cycles 1999 13 1 1 8 10.1029/1998GB900020

[B22] Bouwman AF Lee DS Asman WAH Dentener FJ Van Der Hoek KW Olivier JGJ A global high-resolution emission inventory for ammonia Global Biochem Cycles 1997 11 4 561 587 10.1029/97GB02266

[B23] Sommer SG Hutchings NJ Techniques and strategies for the reduction of ammonia emissions from agriculture Water, Air Soil Pollut 1995 85 237 248 10.1007/BF00483704

[B24] Mosier AR Duxbury JM Freney JR Heinemeyer O Minami K Nitrous oxide emissions from agricultural fields: Assessment, measurement and mitigation Plant and Soil 1996 181 01 10.1007/BF00011296

[B25] Robertson GP Jackson L Nitrogen use efficiency in row-crop agriculture. Crop nitrogen use and crop nitrogen loss Ecology in Agriculture 1997 Academic Press. New York 347 365

[B26] Majundar D Rastogi M Kumar S Pathack M Jain C Kumar U Nitrous oxide emission from an alluvial soil with different nitrogenous fertilizers and nitrogen levels Indian Soc Soil Science 2001 48 732 741

[B27] Brentrup F Kusters K Lammel J Application of the Life Cycle Assessment methodology to agricultural production: an example of sugar beet production with different forms of nitrogen fertilizers European Journal of Agronomy 2001 14 221 233 10.1016/S1161-0301(00)00098-8

[B28] Seabra JEA Macedo IC Balanço de energia e emissões de GEE na produção do açúcar e álcool orgânico na usina São Francisco 2008 http://www.nativealimentos.com.br/upload/Inventario_CO2.pdf

[B29] Gifford RM Stanhill G Energy in different agricultural systems: renewable and non-reneweble sources Energy and Agriculture 1984 Berlim: Springer-Verlag 84 112 Accessed in: 03 dec 2007

[B30] Rezende AS Santos AO Gondim A Xavier RP Coelho CHM Oliveira de OC Alves BJR Boddey RM Urquiaga S EMBRAPA. Efeito Estufa e o Seqüestro de Carbono em Sistemas de Cultivo com Espécies Florestais e na Cultura de Cana-de-açúcar 2001 24 Documento num. 133

[B31] Ripoli TC Molina WF JR Stupiello JP Nogueira MC Saccomano JB Potencial energético de resíduos de cosecha de la cana verde Piracicaba: STAB 1991

[B32] MacCarty N Ogle D Still D Bond T Roden C Willson B Laboratory comparison of the global-warming potential of six categories of biomass cooking stoves Aprovecho Research Center 2007 6

[B33] Ronquim CC Dinâmica espaço temporal do carbono aprisionado na fitomassa dos agroecossistemas do Nordeste do Estado de São Paulo Campinas: Embrapa Monitoramento por Satélite; Ribeirão Preto: ABAGRP, 52 2007 http://www.abagrp.org.br/media/uploads/pdf/lv_fitomassa_nesp.pdf Accessed in: 06 nov 2007

[B34] Ripoli TC Stupiello JP Caruso JGB Zotelli H Amaral JR Efeito da queima na exsudação dos colmos: resultados preliminares 1996 Congresso da sociedade dos técnicos açucareiros e alcooleiros do Brasil, 6, 1996, Maceió. Anais. Piracicaba: STAB

[B35] Gheller ACA Variedades de cana-de-açúcar cultivadas no Estado de São Paulo em 1995 - Censo varietal 1996 Congresso Nacional da Sociedade dos Técnicos Açucareiros e Alcooleiros do Brasil - *STAB*, **6**, Maceió, nov., Anais. Piracicaba: STAB

[B36] Levine J Innes J, Beniston M, Verstraete M Global biomass burning: a case study of the gaseous and particulate emissions released to the atmosphere during the 1997 fires in Kalimantan and Sumatra, Indonesia, in Biomass Burning and its Inter-relationships with the Climate System 2000 Kluwer Academic Publishers: Dordrecht 15 31

[B37] Gullett B Touati A Huwe J Akk A Emissions from Simulated Sugarcane Field Burning Environmental Science &Technology 2006 40 20 PCDD and PCDF 10.1021/es060806k 17120546

[B38] Andrea MO Merlet P Emission of trace gases and aerosols from biomass burning Global Biogeochemical Cycles 2001 15 955 966 10.1029/2000GB001382

[B39] Macedo IC Leal MLRV da Silva Jear Balanço das emissões de gases de efeito estufa na produção e uso do etanol no Brasil Secretaria do Meio Ambiente, Governo de São Paulo 2004 http://www.unica.com.br/download.asp?mmdCode=76A95628-B539-4637-BEB3-C9C48FB29084 Accessed in: 05 nov 2008

[B40] Elia Neto A Nakahodo T Caracterização físico-química da vinhaça 1995 Centro de Tecnologia Canavieira, Piracicaba, São Paulo, Brazil

[B41] Proconve. Control Program of air pollution by vehicles Programa de Controle da Poluição do Ar por Veículos Automotores 2006 http://www.ibama.gov.br/proconve/obter_cagn.php Accessed in: 07 dec. 2008

